# Gamma Knife Radiosurgery for Pituitary Tumors: A Systematic Review and Meta-Analysis

**DOI:** 10.3390/cancers13194998

**Published:** 2021-10-05

**Authors:** Luigi Albano, Marco Losa, Lina Raffaella Barzaghi, Ajay Niranjan, Zaid Siddiqui, John C. Flickinger, Lawrence Dade Lunsford, Pietro Mortini

**Affiliations:** 1 Departments of Neurosurgery and Gamma Knife Radiosurgery, IRCCS Ospedale San Raffaele and Vita-Salute San Raffaele University, 20132 Milan, Italy; barzaghi.linaraffaella@hsr.it (L.R.B.); mortini.pietro@hsr.it (P.M.); 2 Neuroimaging Research Unit, Division of Neuroscience, IRCCS Ospedale San Raffaele and Vita-Salute San Raffaele University, 20132 Milan, Italy; 3 Departments of Neurological Surgery, University of Pittsburgh Medical Center, Pittsburgh, PA 15213, USA; niraax@upmc.edu (A.N.); lunsfordld@upmc.edu (L.D.L.); 4 Center for Image-Guided Neurosurgery, University of Pittsburgh Medical Center, Pittsburgh, PA 15213, USA; siddiquiza@upmc.edu (Z.S.); flickingerjc@upmc.edu (J.C.F.); 5 Department of Radiation Oncology, University of Pittsburgh Medical Center, Pittsburgh, PA 15213, USA

**Keywords:** gamma knife, radiosurgery, pituitary, pituitary adenoma, pituitary tumor, craniopharyngioma

## Abstract

**Simple Summary:**

Pituitary tumors represent approximately 10–15% of all brain neoplasms. Gamma Knife, the most commonly used stereotactic radiosurgery technique worldwide, plays an important role in the treatment of several pituitary neoplasm. It is currently used in cases of residual or recurrent tumors after surgery or as primary treatment when surgery is contraindicated. Its goals are long-term tumor control, preservation of visual function, and, for secreting pituitary adenomas, endocrine remission. Several retrospective case-series (level of evidence IV) on Gamma Knife for pituitary tumors have been published describing encouraging outcomes; only one systematic review and meta-analysis on non-functioning pituitary adenoma has been recently reported. We provide a systematic review of the literature and meta-analysis from the last two decades on Gamma Knife radiosurgery for several pituitary tumors with the aim of describing and confirming safety and effectiveness of this technique.

**Abstract:**

To describe and evaluate outcomes of Gamma Knife radiosurgery (GK) for the treatment of pituitary tumors over the past twenty years, a systematic review and meta-analysis according to PRISMA statement was performed. Articles counting more than 30 patients were included. A weighted random effects models was used to calculate pooled outcome estimates. From 459 abstract reviews, 52 retrospective studies were included. Among them, 18 reported on non-functioning pituitary adenomas (NFPA), 13 on growth hormone (GH)-secreting adenomas, six on adrenocorticotropic hormone (ACTH)-secreting adenomas, four on prolactin hormone (PRL)-secreting adenomas, and 11 on craniopharyngiomas. Overall tumor control and five-year progression free survival (PFS) estimate after one GK procedure for NFPA was 93% (95% CI 89–97%) and 95% (95% CI 91–99%), respectively. In case of secreting pituitary adenomas, overall remission (cure without need for medication) estimates were 45% (95% CI 35–54%) for GH-secreting adenomas, 64% (95% CI 0.52–0.75%) for ACTH-secreting adenomas and 34% (95% CI: 19–48%) for PRL-secreting adenomas. The pooled analysis for overall tumor control and five-year PFS estimate after GK for craniopharyngioma was 74% (95% CI 67–81%) and 70% (95% CI: 64–76%), respectively. This meta-analysis confirms and quantifies safety and effectiveness of GK for pituitary tumors.

## 1. Introduction

Pituitary gland tumors represent approximately 10–15% of all brain neoplasms. Most of them are pituitary adenomas (up to 80–90%), but they include other lesions of different histological nature, both benign and malignant [1,2]. Pituitary neoplasms often are detected because of signs and symptoms related to over- or under-secretion of pituitary gland hormones; others are found because of local compression of nearby structures such as the optic chiasm. Some tumors, however, are detected as incidental findings on magnetic resonance imaging (MRI) or computed tomography (CT) scans performed for some other reasons [1,3].

Treatment options of pituitary tumors include surgery, radiosurgery, radiation therapy, and in the case of hormonally active tumors, medical suppression treatment [1,3,4,5]. For patients with tumors compressing the optic system or those that are hormonally active, therapeutic goals are histological diagnosis, radical removal of the intrasellar lesion to avoid recurrence and relief of any visual impairment or other neurologic symptoms and management of hormonal hypersecretions/deficiencies. Surgery is the first line option for most pituitary tumors except prolactinomas [3,4]; for those tumors found incidentally, surgery is generally indicated for “incidentalomas“ of 1 cm or more in diameter, or when tumor enlargement is detected in patients during serial neuroradiological follow-up [3].

Stereotactic radiosurgery (SRS) is usually employed as an adjuvant treatment in patients with residual or recurrent tumors following surgery. Developments in SRS techniques and their encouraging outcomes have led radiosurgery to become a primary therapy for those where surgery is contraindicated. Gamma Knife radiosurgery (GK) is the most frequently used SRS technique worldwide. The GK system consists of an array of 192 or 201 sources of cobalt-60 that align with an inner collimator to direct the resulting photon beams delivered by the decay of Cobalt 60 (gamma rays). All the beams converge at a single point called the isocenter. GK allows to precisely deliver high doses of radiation to small targets minimizing the volume of normal brain structures irradiated to high doses, such as the optic pathway; it is thus frequently employed in patients with pituitary tumors. GK is usually given in single fraction or, less frequently, in a reduced number of fractions (from 2 to a maximum of 5) [6,7].

Several retrospective case-series and few prospective studies on GK for pituitary tumors have been published describing encouraging outcomes; to our knowledge, a limited number of systematic reviews and meta-analyses on SRS for pituitary tumors have been published, often involving different radiosurgical techniques [8,9,10]. Therefore, the current level of evidence of GK for most pituitary tumors is IV. In this systematic review of the literature and meta-analysis, we mainly focus on GK in the treatment of non-functioning pituitary adenoma (NFPA, namely also null cell adenoma), secreting pituitary adenomas, neurohypophyseal tumors, pituitary carcinomas, and craniopharyngiomas.

## 2. Materials and Methods

**Figure 1:**
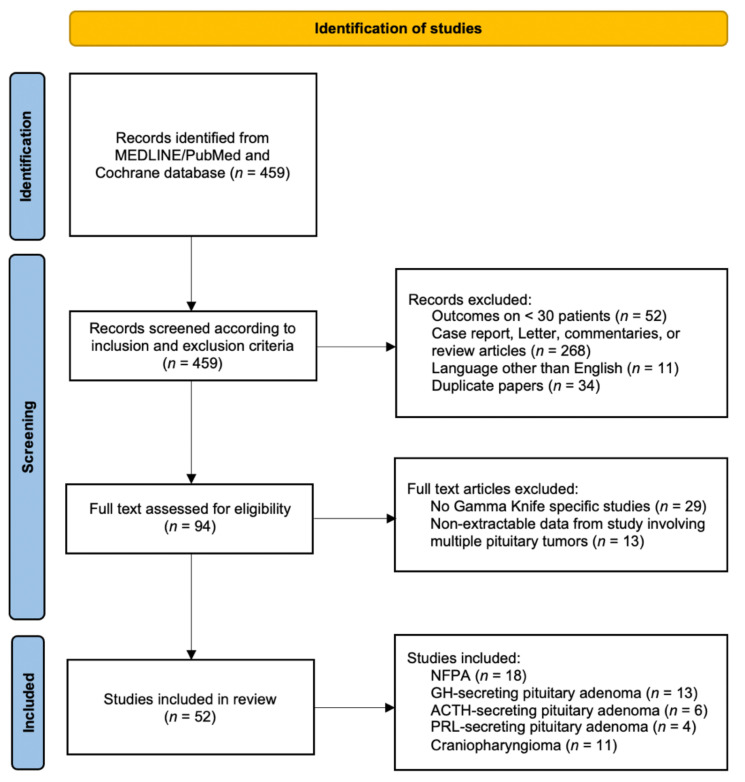
PRISMA Flowchart of study selection. Abbreviations: ACTH = adrenocorticotropic hormone; GH = growth hormone; NFPA = non-functioning pituitary adenomas; PRL = prolactin hormone.

A systematic review of the literature was conducted according to criteria of the Preferred Reporting Items for Systematic Reviews and Meta-analyses (PRISMA). MEDLINE (PubMed) and Cochrane electronic bibliographic database searches were carried out. Furthermore, additional primary research studies were added based on a review of bibliographies of the selected papers. Combinations of the following keywords were used: “gamma knife” OR “radiosurgery” AND “pituitary” AND/OR “adenoma” AND/OR “craniopharyngioma”. Full text articles in the English language published starting from January 2000 up until July 2021 were considered. The initial result identified 459 articles that were subsequently screened. Inclusion criteria accounted for were: retrospective or prospective case series involving at least 30 patients, radiosurgery studies involving the use of GK technique only and description of clinical outcome specific to NFPA, secreting pituitary adenomas, neurohypophyses tumors, pituitary carcinomas, and craniopharyngiomas. On the other hand, the following exclusion criteria were applied: case reports, letters to the editor, commentaries, and expert opinions were excluded; studies involving patients with different pituitary tumors which did not show outcomes clearly divided according to each type of tumor were excluded. As some series included updates on prior studies with inclusion of already published patient cohorts, duplicate papers with similar number of included patients were assessed for any updated data on treatment efficacy or toxicity with the latest report of the largest number of patients included in the final analysis. The outline of search strategy is summarized in Figure 1.

Primary outcome measures from meta-analysis were tumor control (in case of NFPA and craniopharyngioma) and tumor remission (for secreting pituitary adenomas). Secondary outcome measures were 5-year progression-free survival (NFPA and craniopharyngioma), 5-year recurrence-free survival (for secreting pituitary adenomas), and new onset hypopituitarism. RStudio version 1.3.1093 and R package “metafor” were used for meta-analysis, tests for heterogeneity, and analysis of publication bias. The DerSimonian–Laird method was used to assess study variances for overall estimate. Weighted randomized effects models were used to evaluate pooled estimates for primary and second outcomes accounting also for follow-up duration in months, since the studies involved patients with different follow-up. Heterogeneity was assessed through visual inspection of forest plots and using formal tests. The *I^2^* statistics was used to quantify heterogeneity with thresholds of 0%, 25%, 50%, and 75% indicating absent, low, medium, and high heterogeneity, respectively. Funnel plots and Egger test were used for identifying publication bias (Figures S1–S3). A *p*-value of <0.05 was considered significant for all the tests in the analysis. Data are presented with their 95% confidence interval (CI). A narrative evaluation is provided for all other cases.

## 3. Results

The results of the search strategy yielded a total of 459 articles. After a comprehensive review of the published papers, 52 studies met the inclusion criteria for this systematic review. Among them, 18 reported on NFPAs, 13 on growth hormone (GH)-secreting adenomas, six on adrenocorticotropic hormone (ACTH)-secreting adenomas, four on prolactin hormone (PRL)-secreting adenomas, and 11 on craniopharyngiomas. In reference to neurohypophysis tumors and pituitary carcinomas, no articles met the inclusion criteria; few case reports or small case series are currently reported in medical literature probably due to the rarity of these tumors. All included studies were retrospective in nature; most of them (*n* = 45, 87%) are single institutional case-series, whereas seven studies (13%) are from multi-institutional partnerships (six from health centers in the United States and one from multiple institutions in Japan).

### 3.1. Non-Functioning Secreting Pituitary Adenoma

**Figure 2:**
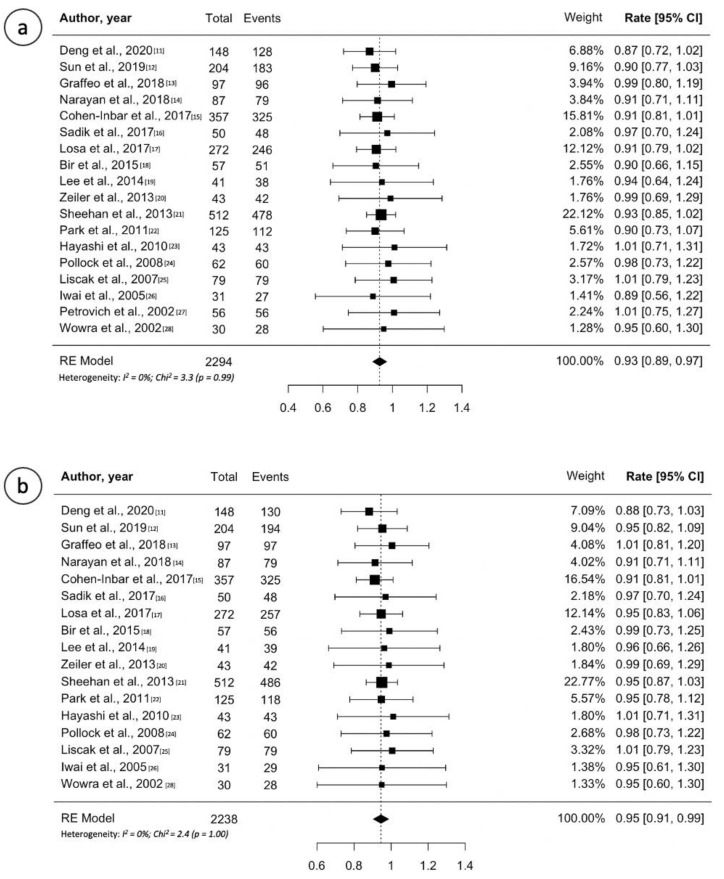
(**a**) Forest plot of overall tumor control following Gamma Knife treatment for non-functioning pituitary adenomas; (**b**) Forest plot of 5-year progression free survival after Gamma Knife treatment for non-functioning pituitary adenomas. Random effects models pooled estimates are presented and heterogeneity analysis are included.

Study details, treatment overview and outcomes are reported in Table 1 [11,12,13,14,15,16,17,18,19,20,21,22,23,24,25,26,27,28]. Across all 18 papers, the median number of patients treated in single institutional case series was 57 (range, 30–272 patients). The median follow-up reported was 48 months (range, 35–86 months). Furthermore, the median marginal dose was 15 Gy (range, 12–20 Gy). The majority of studies (*n* = 13, 72%) showed tumor control rates at last follow-up ranging between 90% and 100%. Based on the pooled analysis, 2119 of 2294 patients (0.93, 95% CI 0.89–0.97; I^2^ = 0%, *p* = 0.99) from 18 studies had local tumor control (Figure 2a). All studies but one (94%) described the five-year progression-free survival (PFS) ranging 90–100% and 10 of them reported a five-year PFS ≥ 95%. Random effects meta-analysis for five-year PFS are shown in Figure 2b, with estimates of 95% (95% CI: 91–99%; I^2^ = 0%, *p* = 1.00). Only six studies reported the 10-year PFS ranging 74–92% [11,12,17,18,19]. Notably, referring to tumor volume decrease after GK, the majority of studies reported a rate tumor shrinkage of at least 50% (range, 25–83%) over time. New-onset hypopituitarism ranged 0–32%. Random effects meta-analysis for new hypopituitarism is shown in Figure S4, with estimates of 18% (95% CI: 13–23%; I^2^ = 71%, *p* < 0.001). The incidence of radiation induced optic neuropathy ranged between 0% and 7%.

### 3.2. GH-Secreting Pituitary Adenoma

**Figure 3:**
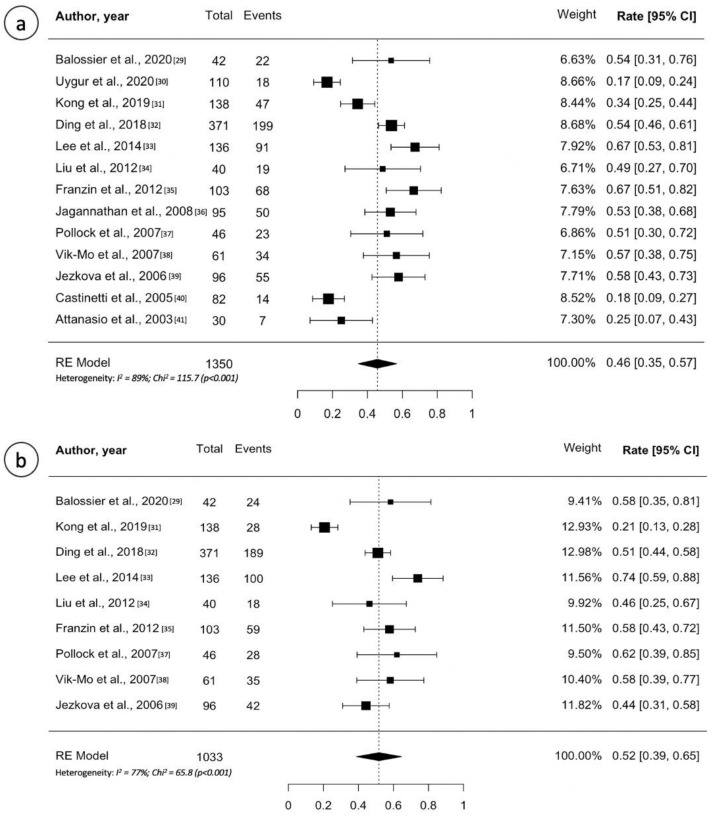
(**a**) Forest plot of overall tumor control following Gamma Knife treatment for growth hormone-secreting pituitary adenomas; (**b**) Forest plot of 5-year recurrence-free survival after Gamma Knife treatment for growth hormone-secreting pituitary adenomas. Random effects models pooled estimates are presented and heterogeneity analysis are included.

Table 2 lists all studies on GH-secreting adenomas involved in this review and their outcomes [29,30,31,32,33,34,35,36,37,38,39,40,41]. The median number of patients included in single institutional studies was 95 (range, 30–138 patients) followed up for a median of 67 months after GK treatment (range, 49–166 months). The median marginal dose delivered to the tumor edge ranged between 20 and 28 Gy. As shown in Table 2, criteria of cure in patients with acromegaly treated by GK includes normalization of age appropriate insulin-like growth factor 1 (IGF1) and/or GH levels; the latter varies study by study. Most series considered a cut-off of 2.5 µg/L, others proposed a cut-off of 1 µg/L whereas some authors took into account the oral glucose tolerance test (OGT). Despite this mismatch in the criteria of hormonal remission, in 8 of 13 included studies (62%) the remission rate ranged 50–65%. The five-year recurrence-free survival (RFS) ranged from 20% to 73%. Random effects meta-analysis for overall remission and five-year RFS are shown in Figure 3, with estimates of 46% (95% CI: 35–57%; I^2^ = 89%, *p* < 0.001) and of 52% (95% CI: 41–60%; I^2^ = 77%, *p* < 0.001), respectively. Few studies (4 out of 13) reported the 10-year RFS (Table 2) [31,32,35,38]. The multicenter study involving the largest cohort of patients (*n* = 371) showed a 10-year RFS of 69% [32]. New-onset of a reduction in at least one pituitary hormonal axis (hypopituitarism) ranged between 0% and 40%. Random effects meta-analysis for new hypopituitarism is shown in Figure S4, with estimates of 20% (95% CI: 14–27%; I^2^ = 87%, *p* < 0.001). The incidence of radiation induced optic neuropathy ranged between 0% and 5%.

### 3.3. ACTH-Secreting Pituitary Adenoma

**Figure 4:**
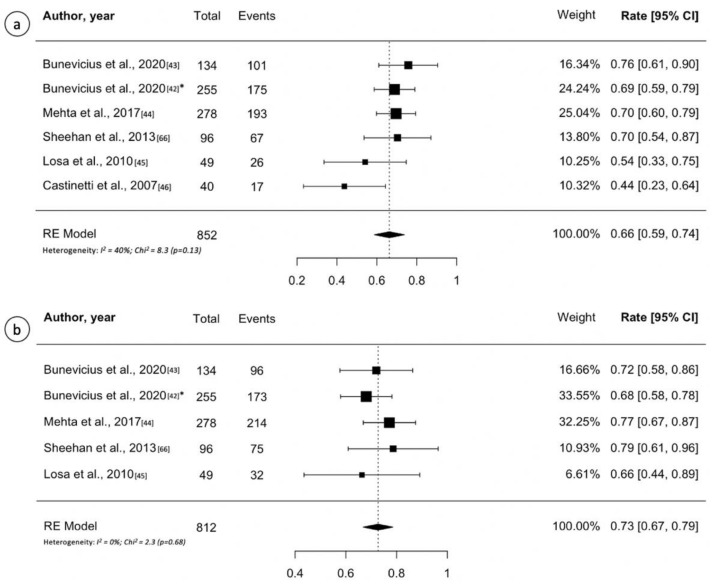
(**a**) Forest plot of overall tumor control following Gamma Knife treatment for adrenocorticotropic hormone-secreting pituitary adenomas; (**b**) Forest plot of 5-year recurrence-free survival after Gamma Knife treatment for adrenocorticotropic hormone-secreting pituitary adenomas. Random effects models pooled estimates are presented and heterogeneity analysis are included. * Multicenter study.

Study details, patient characteristics and treatment outcomes are reported in Table 3 [42,43,44,45,46]. The number of patients evaluated ranged between 40 and 278. The median marginal dose ranged between 22 and 29.5 Gy. The criterion of normal 24-hour urinary free cortisol (UFC) concentration off cortisol lowering medications is universally adopted in all GK series. Most authors also require additional criteria, such as normal basal ACTH and/or suppression of cortisol secretion during the low-dose dexamethasone test (LDDST). Remission of hypercortisolism after GK occurs in more than 50% of cases in four of the five included papers (80%). Notably, the study with the lowest remission rate (43%) adopted both UFC and LDDST as criterion of cure (Table 3) [46]. Based on the pooled analysis, 579 of 852 patients (0.66, 95% CI 0.59–0.74; I^2^ = 40%, *p* = 0.13) from six studies had tumor remission (Figure 4a). The five-year RFS ranged from 66% to 78%. Random effects meta-analysis for five-year RFS are shown in Figure 4b, with estimates of 73% (95% CI: 67–79%; I^2^ = 0%, *p* = 0.68). Only the study by Mehta et al., accounting for the widest cohort of ACTH-secreting pituitary adenoma patients (*n* = 278), reported the 10-year RFS (80%) [44]. Recurrence of disease after apparent remission of hypercortisolism ranged 16–20%. New-onset hypopituitarism ranged 15–36%. Random effects meta-analysis for new hypopituitarism is shown in Figure S4, with estimates of 28% (95% CI: 22–34%; I^2^ = 58%, *p* = 0.048). The incidence of radiation induced optic neuropathy ranged 0–2%.

### 3.4. PRL-Secreting Pituitary Adenoma

**Figure 5:**
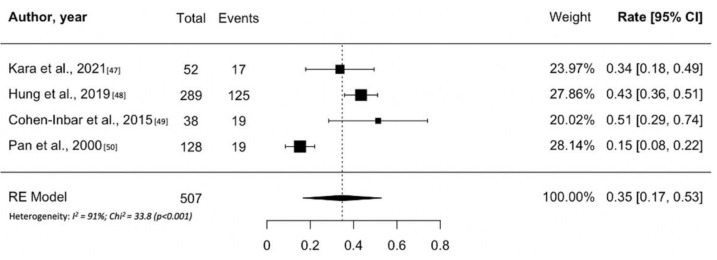
Forest plot of overall tumor control following Gamma Knife treatment for prolactin hormone-secreting pituitary adenomas.

Only four studies on GK treatment for prolactinomas were included in this systematic review and meta-analysis (Table 4) [48,49,50,51]. Typically such patients have larger and regionally invasive tumors with PRL levels that indicate cavernous sinus invasion. The number of patients ranged between 38 and 289, with the latter reported in a multi-institutional study. Follow-up ranged between 13 and 45 months and the median marginal dose between 17 and 31 Gy. In contrast to other secreting adenomas, normalization of PRL levels was the only criterion used by all studies to define the success of GK (Table 4). The remission of prolactinomas after GK treatment ranged between 15% and 50%. Notably, the study reporting remission rate of 15% included patients treated with GK as primary therapy [51]. However, random effects meta-analysis for remission of hyperprolactinemia are shown in Figure 5, with estimates of 35% (95% CI: 17–53%; I^2^ = 91%, *p* < 0.001). Only the multi-institutional study by Hung et al. reported the five-year RFS (41%) [49]; no pooled analyses were thus possible. Recurrence of hyperprolactinemia after hormonal remission occurs uncommonly; in the two larger studies, 8% and 5% of patients had a recurrence of disease. No studies showed the 10-year RFS. New-onset hypopituitarism ranged 19–26%. Many patients may require long term hormonal suppression using agents such as dostinex or cabergoline. Random effects meta-analysis for new hypopituitarism is shown in Figure S4, with estimates of 24% (95% CI: 19–29%; I^2^ = 0%, *p* = 0.74). The incidence of radiation induced optic neuropathy ranged 3–4%.

### 3.5. Craniopharyngioma

**Figure 6:**
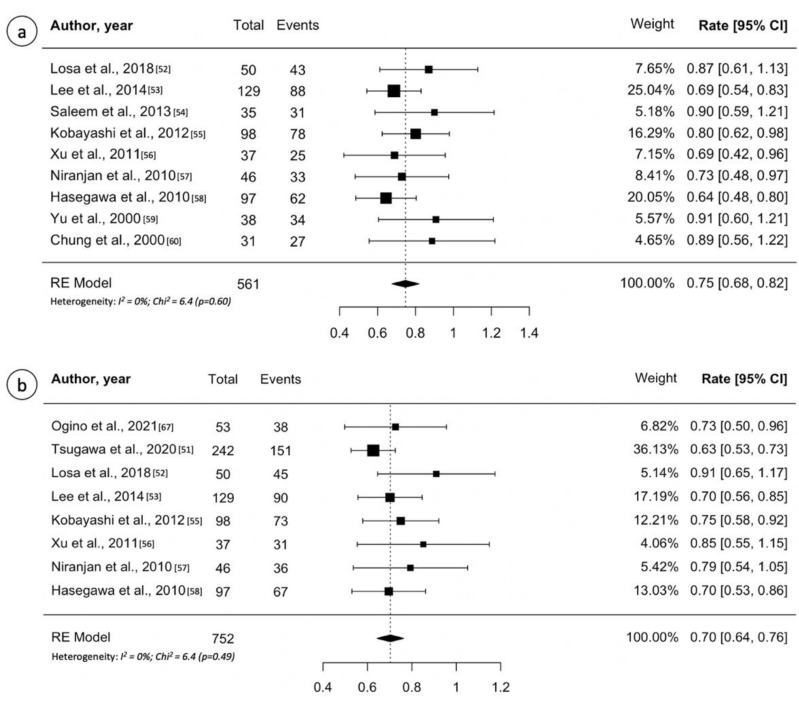
(**a**) Forest plot of overall tumor control following Gamma Knife treatment for craniopharyngioma; (**b**) Forest plot of 5-year recurrence-free survival after Gamma Knife treatment for craniopharyngioma. Random effects models pooled estimates are presented and heterogeneity analysis are included.

Table 5 lists all studies on GK treatment for craniopharyngioma included in this review [52,53,54,55,56,57,58,59,60,61,62]. Across all 11 papers, the median number of patients treated in single institutional case series was 48 (range, 31–137 patients). The median follow-up reported was 61 months (range, 16–118 months) and the median marginal dose 12 Gy (range, 11–14 Gy). The reported local tumor control rate after one or more GK procedures ranged between 68% and 90%. Based on the pooled analysis, 421 of 561 patients (0.75, 95% CI 0.68–0.82; I^2^ = 0%, *p* = 0.60) from 11 studies had overall tumor control (Figure 6a). On the contrary, all studies reported a five-year PFS > 60% (range, 62–90%). Random effects meta-analysis for five-year PFS are shown in Figure 6b, with estimates of 70% (95% CI: 64–76%; I^2^ = 0%, *p*= 0.49). The 10-year PFS ranged between 43% and 78%. Referring to treatment-related toxicity, new-onset hypopituitarism is lower than those reported for pituitary adenomas treatment probably because most patients already have hypopituitarism and diabetes insipidus at the time of GK. It ranged 0–20%, whereas the rate of radiation induced optic neuropathy ranged 0–5%.

## 4. Discussion

### 4.1. Gamma Knife Outcome for Non-Functioning Pituitary Adenoma

The principal aim of GK in patients affected by NFPA is tumor control (prevention of tumor growth requiring additional surgical or radiosurgical care). This allows to reduce the risk of regrowth after incomplete surgical resection and/or tumor recurrence. Although actual level IV evidence (all of the studies, both single and multi-institutional, are retrospective case series), GK was observed to be an effective treatment for patients with NFPA. In fact, the pooled estimate analysis estimated a 93% of overall tumor control. Furthermore, most of studies reported a five-year PFS ≥ 95%. On the contrary, long-term tumor control was rarely reported. However, available data show an estimated loss of tumor control at 10 years from treatment (Table 1).

Large target volume (>4.5 cc according to Park et al. [22] or >5 cc according to Narayan et al. [14]) and suprasellar extension relate to an unfavorable GK outcome in the multicenter study by Sheehan et al. [21], whereas a history of multiple surgical procedures for pituitary adenoma was the only significant factor of poor outcome in another study [17].

In reference to adenoma relapse, as stated by Losa et al., tumor recurrence often represents a new growth located outside the field of previous irradiation (“out of field”), probably not visible at the time of pre-treatment MRI; less commonly relapses of adenoma appear in the field of irradiation and represents primary failure of GK to control the treated lesion [17].

Most of the patients were treated with a median marginal dose of 15 Gy, ranging from 12 to 20 Gy. Commonly, the choice of marginal dose is based on the maximum one delivered to the anterior optic pathway. However, no significant differences in tumor control rate have been observed using greater prescription dose. On the other hand, the lowest effective dose remains controversial. Mingione et al., reported a minimal effective dose of 12 Gy and stated that doses greater than 20 Gy did not lead any improvement on tumor control [63].

Until recently, one of the most controversial issues was the timing of GK after surgical debulking of the tumor. Currently, there is a radiosurgical consensus to recommend early radiosurgical treatment in patients who underwent resection and have clear residual tumor. Pomeraniec et al. recently compared clinical outcome of patients treated with early SRS versus those who received SRS after more than 6 months from surgery. The authors described lower risk of imaging and symptomatic tumor progression in the former group of patients [64].

Due to its high tumor control rates, GK may be used as a primary treatment in selected patients with high surgical risk comorbities or patient refusal. Several studies included patients who received GK as the primary management [6]. Lee et al., in particular, described a total of 41 patients with NFPA who underwent GK as primary treatment; they reported 5- and 10-year PFS of 94% and 85%, respectively, in line with the other series [19].

### 4.2. Gamma Knife Outcome for Secreting Pituitary Adenoma

Unlike NFPA, the additional goal of GK in patients with secreting pituitary adenomas is normalization of hormone hypersecretion. GK is typically used as an adjuvant management in patients with persistent acromegaly, Cushing’s disease and invasive medically recalcitrant and recurrent prolactinomas that remain symptomatic after one or more failed operations. Higher marginal doses are required for hormonally secreting pituitary adenomas. The most effective normalization of hormone hypersecretion is, in fact, reported when doses from 20 to 25 Gy at the tumor margin are used. In GK planning the maximal tumor dose is often two or more times the tumor margin or edge dose. Validation of universal criteria of cure is currently lacking, which may account for various discrepancies reported in the medical literature.

In reference to acromegaly, criteria of cure in patients treated by GK includes mainly normalization of insulin-like growth factor 1 (IGF1) and/or GH levels (typically < 1µg/L). Despite the variability in the criteria of hormonal remission, a majority of studies on GK for acromegaly reported remission rate that ranged between 50% and 65%. However, three studies described a remission rate less than 25% [30,40,41]. The pooled estimate analysis showed 46% of remission rate. Notably, the probability of endocrine remission gradually increased over the years, reaching a plateau at 10–12 years after GK approximating by 70% at 10 years (Table 2). In patients with GH-secreting adenoma, some studies found a negative association between somatostatin analog (SSA) use at the time of GK and the effects of radiosurgery, whereas others failed to find a significant relationship [6,65,66,67]. However, almost all studies found a trend towards worse results in patients taking SSA than in untreated patients, or in those who had quitted medical treatment while waiting for GK. In this context, the largest series published to date by Ding et al. confirmed a negative impact of concomitant use of growth hormone production agents such as sandostatin [32]. While a “radioprotective” effect of drugs acting directly at the pituitary level may be hypothesized, any negative effect in patients taking pegvisomant, which exerts its effects by suppression of the liver pathway manufacturing IGF 1, is unclear. Further research in this area would be necessary.

In patients with Cushing’s disease, the criterion of UFC concentration on cortisol lowering medications is universally adopted in all GK series; however, some authors also require additional criteria, as reported in Table 3. In contrast to other secreting pituitary adenomas, a relevant issue affecting patients with Cushing’s disease is the absence of a clear visible tumor on neuroimaging. In fact, the percentage of patients with no clear pituitary lesion on magnetic resonance imaging (MRI) may be as high as 30–40%. In such patients, whole sellar GK has been suggested in order to obtain a “radiosurgical hypophysectomy” despite the potential for endocrine axis losses [47]. Remission of hypercortisolism occurs in more than 50% of cases treated by GK. Random effects meta-analysis estimates remission and five-year RFS of 66% and 73%, respectively. Notably, the study by Castinetti et al. with the lowest remission rate is the one requiring a normal LDDST as a criterion of cure [46]. In contrast to GH-secreting adenomas, most of the remissions occur within three to four years from GK. A difference in radiosensitivity between the two types of secreting pituitary adenoma is, therefore, suggested [47]. Furthermore, in ACTH-secreting adenomas, the use of cortisol lowering medications, especially ketoconazole, has been associated with an unfavorable GK outcome or with a slower time of hormone normalization [46,47]. Since ketoconazole is prescribed to reduce the adrenal manufacturing of cortisol, several authors suggest quitting antisecretory medications before GK [46]. Patients receiving GK on the whole sellar, due to a lack of a discrete tumor on pretreatment MRI, seem to have the same probability of remission as those with a visible tumor target [47].

In contrast to other secreting adenomas, normalization of PRL levels was the only criterion used by all studies to define the success of GK (Table 4). Considering the other subtypes of pituitary adenomas, the efficacy of GK is much less in patients with invasive prolactinomas. The probability of obtaining normalization of remission is, in fact, generally lower than 50%. Interestingly, the remission rate between the larger multicenter studies by Hung et al. is similar to that reported in smaller series [49]. The probability of normalizing PRL levels by combining GK and dopamine agonists approaches 50–70% at five years [39,49,50]. A lower PRL level before GK (a sign of less tumor invasiveness of regional structures such as the cavernous sinus) is associated with better hormonal remission in several studies [34,49].

### 4.3. Gamma Knife Outcome for Craniopharyngioma

Surgical resection remains the optimal treatment for craniopharyngiomas since gross total tumor removal is associated with the best long-term overall and recurrence-free survival. Gross total resection is often associated with panhypopituitarism that requires replacement of both anterior and posterior pituitary function (diabetes insipdus). Caniopharyngiomas are often adherent to critical brain and vascular structures, so that residual and recurrent tumors are frequent even after skilled microsurgical or endoscopic procedures. The rate of gross total resection varies between 59% and 90% [62]. GK is employed for the treatment of residual or recurrent craniopharyngioma. According to our review the 5- and 10-year PFS ranged 62–90% and 43–78%, respectively. The pooled estimate analysis showed 75% of overall local tumor control and 70% of five-year PFS. Overall survival ranging from 91.5% to 97% at five years and from 82% to 91% at 10 years after GK. Smaller tumor volume, higher margin dose and greater percentage of tumor receiving at least 12 Gy are reported as factors associated to better tumor control [62]. The studies reported the highest marginal dose were, in fact, those associated with the best tumor control rate and PFS over time. Due to the basal anatomical location of these tumors and their close proximity/contact to the optic system, dose reduction to decrease the risk of new or further optic neuropathy may be necessary [7]. Losa et al. suggested that hypofractionated GK is a safe and effective treatment allowing to prescribe high radiation dose to the tumor minimizing the risk of radiation-induced optic injury as well (see 4.4 Multisession Gamma Knife radiosurgery for pituitary tumors) [53]. Recently, Ogino et al. found that when ≥85% of tumor volume receives 12 Gy or greater, tumor control can be maximized while reducing the risk of optic nerve injury [62].

### 4.4. Complications

#### 4.4.1. Hypopituitarism

The most common delayed side effect of GK for pituitary tumors is new onset hypopituitarism. The pooled estimate of new onset hypopituitarism was 18% in NFPA and ranged 20–28% in hormone-secreting adenomas. With regards to craniopharyngioma, it is lower than those reported for pituitary adenomas probably because most patients already have hypopituitarism at the time of GK. Notably, a clear time point to compare this complication among included studies is not possible to determine. Thyroid function was affected the most, followed by alterations of gonadotrophic hormone, ACTH, and GH. Several factors were associated with the risk of new onset hypopituitarism such as age, duration of follow-up, marginal dose to the tumor, disease extension, suprasellar extension [68,69]. The maximum dose received by infundibulum and pituitary stalk seems to be the most important factor [6]. Suprasellar extension, for example, correlates to new onset hypopituitarism due to unavoidable proximity to infundibulum, sometimes not visible on MRI, and subsequently the high dose it receives. On the contrary, Hayashi et al., report a case series of patients with NFPA treated in the cavernous sinus only, and did not report any new onset endocrinological complication [23]. However, to date, a cut-off dose below which the patient will be not affected by hypopituitarism after GK does not exist.

#### 4.4.2. Optic Neuropathy

Most GK studies showed an optic nerve radiation induced neuropathy rate of less than 5%. To date, in the field of radiosurgery a maximum point dose of 10–12 Gy in single fraction to the optic apparatus is widely accepted and is in line with the dose tolerance reported by many case series. Furthermore, a recent meta-analysis on dosimetric and clinical predictors of radiation induced optic injury after stereotactic radiosurgery reported a risk of optic neuropathy <1% with optic apparatus maximum point doses <10 Gy in a single fraction [70]. The majority of cases of optic neuropathy and oculomotor damage occurred in patients who had already received radiotherapy in the past. Previous radiotherapy, either conventional or radiosurgery, increases thus the risk of optic injury; it is further affected by prior dose and fractionations, as well as duration between radiotherapy courses [70]. Therefore, when deciding whether GK can be prescribed in patients with any history of radiotherapy, it is mandatory to know the dose of radiation absorbed by the optic pathway during the previous treatment and to maintain the total dose of radiation within acceptable safety limits.

#### 4.4.3. Other Rare Toxicities

Some transient side effects were reported to be related to the frame placement such as headache, pin site dysesthesias/swelling. Cranial nerve neuropathies causing oculomotor defects ranged between 2% and 3%. Hayashi et al., described two cases of oculomotor nerve palsy out of 89 patients (2%) treated with GK for pituitary adenomas extending to cavernous sinus; in both cases, cumulative maximum dose to the cavernous sinus was possibly >40 Gy and symptoms resolved with steroid therapy [23]. Occlusion of the intracavernous carotid artery as result of irradiation was sporadically reported even in patients treated for a hormone-secreting pituitary adenoma [71,72]. Most cases were neurologically asymptomatic probably because occlusion of the carotid artery occurred progressively over several years, thus allowing the efficient development of collateral blood circulation. However, the wall of the intracavernous carotid should be added to the organs at risk during the planning of GK to avoid hot spots of radiation in the proximity of the carotid artery. Cases of radiation necrosis after GK for pituitary tumors was rarely reported [6].

### 4.5. Multisession Gamma Knife Radiosurgery for Pituitary Tumors

As stated above, GK for pituitary tumors is traditionally delivered in a single session, using marginal doses of 12–30 Gy, with the major concern of tumor control/hormonal remission. When the optic pathway is too close to the tumor margin, the risk of optic injury is increased. For this reason, fractionated stereotactic radiosurgery (two to five sessions) has been recently proposed to combine advantages of conventional radiation therapy and SRS [6]. A previous technique for fractionation, still utilized in arteriovenous malformation treatment, is volume staging, where different regions of the target are treated to the full dose across multiple session. More recently hypofractionated stereotactic radiation has been explored, with multiple stereotactic deliveries to the full target volume across three to five sessions with a lower prescription dose. This strategy leads to a reduction of the volume of normal tissue that receives high radiation doses, achieving delivery of an effective radiosurgical dose to pituitary tumor [73]. However, major studies of this technique for the treatment of tumors of the sellar region are lacking, probably because of its recent introduction [74,75]. In this context, the recently introduced Gamma Knife Icon model should facilitate a hypofractionated radiosurgical approach. The larger retrospective case series of 47 patients with pituitary adenomas who underwent fractionated GK (three fractions) showed a tumor control rate of 100% at a mean follow-up of around four years. A median prescription dose per fractions was 7 Gy (range, 6.5–13) [73]. Losa et al. compared single fraction versus multi-fractions GK in patients with craniopharyngioma. No significant differences in terms of treatment effectiveness were reported between the two groups and therefore GK seems a very promising treatment also in patients with large residual or recurrent craniopharyngioma [53]. Although promising, future prospective studies are needed to better validate the effectiveness of this technique. The use standard fractionated radiation therapy (25–30 fractions at 1.8–2 Gy per fraction) has been reserved for cases of circumferential/bulky optic structure involvement not amenable to separation surgery. Fractionated radiation therapy likely leads to high endocrine axis losses over time, reduced efficacy compared to radiosurgery, and higher risk of delayed adjacent late tumor development.

### 4.6. Other Pituitary Tumors

According to inclusion and exclusion criteria, no other intrinsic pituitary tumors were included in our review. Two case reports on GK treatment for pituitary carcinomas currently exist in medical literature [76,77]. However, because of the rarity of these lesions, neither treatment outcomes nor management can be fully defined.

A case series on GK treatment for pituitary spindle cell oncocytomas has been published [78]. The authors reported five patients treated with GK after previous transsphenoidal surgery (median margin dose 12 Gy, range 12–14 Gy). No tumor volume progression or treatment side effects were described at last follow-up (mean 52 months).

### 4.7. Methodological Considerations and Limitations

When interpreting the results of this meta-analysis, several factors call for consideration. The heterogeneity of marginal dose and treatment planning, inevitably slightly different from one group to another, introduces variability. Furthermore, retrospective studies are known to introduce potential recall bias. Particular attention should also be paid to different criteria of cure for secreting pituitary adenomas adopted by included studies. Ideally, all studies should have used the same criteria, with comparable remission rates and recurrence rates between all study cohorts. As another potential weakness, follow-up time-points were not homogeneous between cohorts. Although the follow-up between cohorts included in the meta-analysis may differ, our analysis with follow-up as a nuisance factor did not provide evidence that this significantly influenced the effect size in any category studied.

A new WHO classification of pituitary tumors has been published; however, no studies focused on radiosurgery for pituitary tumors according to the latest histological classification have been reported. For future studies on SRS, that variability in the classification of pituitary tumors should be considered.

## 5. Key Takeaways

- A margin dose of 12–15 Gy is used for nonfunctioning pituitary adenomas;
- Higher margin doses (up to 20–30 Gy) are used for functional adenomas;
- GK SRS is safe and provides tumor control in >90% patients with recurrent or residual nonfunctioning pituitary adenomas;
- Risks of visual dysfunction, or neurological deficit appear to be quite low;
- Delayed Endocrinopathy can be expected in 30–40% patients;
- The endocrine remission response to SRS is best with ACTH producing tumors, followed by GH producing tumors, with prolactinoma having the poorest response.

## 6. Conclusions

GK radiosurgery plays a crucial role as adjuvant treatment of patients with pituitary tumors or as primary treatment when surgery is contraindicated. Our results confirm its effectiveness. The multidisciplinary approach of GK remains the key strength to better define optimal indications and treatment planning. Collaborations among GK centers worldwide as well as current progresses in neuroimaging, technology, dose planning, tumor histology, and molecular analyses could lead to improved results, new knowledge, and expansion of indication of GK for pituitary tumors.

## Figures and Tables

**Table 1:** Non-functioning pituitary adenoma Gamma Knife treatment outcomes and toxicities.

Author	Year	No.	Median Dose (Gy)	Median FU (Months)	Overall Tumor Control (%)	PFS (5-y)	PFS (10-y)	Tumor Shrinkage (%)	New Hypopituitarism (%)	Optic Neuropathy
Deng et al. [11]	2020	148	14	65	87%	88%	74%	75%	28%	4%
Sun et al. [12]	2019	204	14	86	90%	95%	92%	50%	18%	2.5%
Graffeo et al. [13]	2018	97	15	48	99%	100%	NR	52%	31%	0%
Narayan et al. [14]	2018	87	15	48	91%	91%	NR	54%	21%	0%
Cohen-Inbar et al. [15]	2017	357	14	40	91%	91%	NR	80.5%	4%	1%
Sadik et al. [16]	2017	50	15	40	95%	95%	NR	24%	22%	0%
Losa et al. [17]	2017	272	15	79	90%	95%	79%	NR	NR	NR
Bir et al. [18]	2015	57	15	46	90%	98%	90%	56%	19%	3.5%
Lee et al. [19] *	2014	41	12	48	93%	94%	85%	83%	25%	0%
Zeiler et al. [20]	2013	43	14	35	98%	98%	NR	51%	NR	2%
Sheehan et al. [21]	2013	512	16	36	93%	95%	85%	NR	21%	7%
Park et al. [22]	2011	125	13	62	90%	94%	76%	53%	24%	2%
Hayashi et al. [23]	2010	43	18	36	100%	100%	NR	64%	0%*	0%*
Pollock et al. [24]	2008	62	16	64	95%	95%	NR	60%	32%	0%
Liscak et al. [25]	2007	79	20	60	100%	NR	NR	NR	14%	0%
Iwai et al. [26]	2005	31	14	60	87%	93%	NR	58%	7%	0%
Petrovich et al. [27]	2002	56	15	36	100%	NR	NR	NR	4%	0%
Wowra et al. [28]	2002	30	16	58	93%	93%	NR	NR	14%	0%

* Only cavernous sinus location; abbreviations: FU = follow-up; Gy = gray; No = number; NR = not reported; PFS = progression-free survival; y = year.

**Table 2:** GH-secreting pituitary adenoma Gamma Knife treatment outcomes and toxicities.

Author	Year	No.	Median Dose (Gy)	Median FU (Months)	Remission Rate (%)	Recurrence (%)	Hormonal Criteria	RFS (5-y)	RFS (10-y)	Tumor Shrinkage (%)	New Hypopituitarism (%)	Optic Neuropathy
Balossier et al. [29]	2020	42	28	60.5	52%	NR	IGF-1	57% at 7 years	NR	36%	19%	5%
Uygur et al. [30]	2020	110	23^	78	16%	NR	IGF-1; GH < 1 µg/L	NR	NR	94%	5%	NR
Kong et al. [31]	2019	138	25	85^	34%	NR	IGF-1; GH ≤ 2.5 µg/L	20%	45%	NR	9%	NR
Ding et al. [32]	2018	371	24^	79^	54%	9%	IGF-1	51%	69%	65%	26%	3.5%
Lee et al. [33]	2014	136	25	61.5	65%	8%	IGF-1; OGT-GH < 1 µg/L	73% at 6 years	NR	47%	32%	3%
Liu et al. [34]	2012	40	21	72	48%	NR	IGF-1; GH < 2.5 µg/L	45%	NR	68%	40%	0%
Franzin et al. [35]	2012	103	21.5	71	61%	3%	IGF-1; GH < 2.5 µg/L	58%	80%	43%	8%	0%
Jagannathan et al. [36]	2008	95	22	57^	53%	NR	IGF-1	NR	NR	92%	34%	4%
Pollock et al. [37]	2007	46	20	63	50%	NR	IGF-1; GH < 2 µg/L	60%	NR	70%	33%	0%
Vik-Mo et al. [38]	2007	61	26	67^	57%	NR	IGF-1	58%	86%	NR	23%	NR
Jezkova et al. [39]	2006	96	NR	54	57%	NR	IGF-1; OGT-GH < 1 µg/L	44%	NR	62%	32%	0%
Castinetti et al. [40]	2005	82	26	49	17%	NR	IGF-1; GH < 2 µg/L	NR	NR	NR	17%	NR
Attanasio et al. [41]	2003	30	20	46	23%	NR	IGF-1; GH < 2.5 µg/L	NR	NR	37%	0%	2%

^ Mean; abbreviations: FU = follow-up; GH = growth hormone; IGF-1 = insulin like growth factor 1; Gy = gray; No = number; NR = not reported; OGT = oral glucose tolerance test; PFS = progression-free survival; RFS = recurrence-free survival; y = year.

**Table 3:** ACTH-secreting pituitary adenoma Gamma Knife treatment outcomes and toxicities.

Author	Year	No.	Median Dose (Gy)	Median FU (Months)	Remission Rate (%)	Recurrence (%)	Hormonal Criteria	RFS (5-y)	RFS (10-y)	Tumor Shrinkage (%)	New Hypopituitarism (%)	Optic Neuropathy
Bunevicius et al. [43]	2020	134	22 ^	64	75%	18%	UFC; cortisol	72%	NR	53%	35%	2%
Bunevicius et al. [42] *	2020	255	23 ^	66 ^	69%	18%	UFC; cortisol	68%	NR	41%	26%	2%
Mehta et al. [44]	2017	278	24 ^	51	69%	18%	UFC	77%	80%	NR	25%	1%
Shehaan et al. [47]	2013	96	22 ^	48	70%	16%	UFC; cortisol	78%	NR	70%	36%	2%
Losa et al. [45]	2010	49	25	48	53%	NR	UFC	66%	NR	NR	NR	NR
Castinetti et al. [46]	2007	40	29.5	48	43%	NR	UFC; LDDST	NR	NR	NR	15%	0%

* Multicenter study; ^ Mean; abbreviations: FU = follow-up; Gy = gray; No = number; NR = not reported; LDDST = low-dose dexamethasone suppression test; PFS = progression-free survival; RFS = recurrence-free survival; UFC = urinary free cortisol; y = year.

**Table 4:** PRL-secreting pituitary adenoma Gamma Knife treatment outcomes and toxicities.

Author	Year	No.	Median Dose (Gy)	Median FU (Months)	Remission Rate (%)	Recurrence Rate (%)	Hormonal Criteria	RFS (5-y)	RFS (10-y)	Tumor Shrinkage (%)	New Hypopituitarism (%)	Optic Neuropathy
Kara et al. [48]	2021	52	17	13	33%	NR	Normal PRL	NR	NR	69%	19%	4%
Hung et al. [49]	2019	289	22	43	43%	NR	Normal PRL	41%	NR	NR	25%	3%
Cohen-Inbar et al. [50]	2015	38	25	42	50%	NR	Normal PRL	NR	NR	NR	26%	NR
Pan et al. [51]	2000	128	31 ^	45 ^	15%	NR	Normal PRL	NR	NR	NR	NR	NR

^ Mean; abbreviations: FU = follow-up; Gy = gray; No = number; NR = not reported; PFS = progression-free survival; PRL = prolactin; RFS = recurrence-free survival; y = year.

**Table 5:** Craniopharyngioma Gamma Knife treatment outcomes and toxicities.

Author	Year	No.	Median Dose (Gy)	Median FU (Months)	Overall Tumor Control (%)	PFS (5-y)	PFS (10-y)	Tumor Shrinkage (%)	New Hypopituitarism (%)	Optic Neuropathy
Ogino et al. [62]	2021	53	12	118	NR	72%	53%	NR	2%	2%
Tsugawa et al. [52]	2020	242	11.4 ^	61	NR	62%	43%	NR	9%	2%
Losa et al. [53]	2018	50	14.3 ^	75 ^	86%	90%	78%	64%	20%	2%
Lee et al. [54]	2014	137	12	46	69%	70%	44%	54%	8%	1%
Saleem et al. [55]	2013	35	11.5	22	88%	NR	NR	NR	0%	NR
Kobayashi et al. [56]	2012	100	11.5	65	80%	74%	60%	NR	NR	NR
Xu et al. [57]	2011	37	14.5	50	68%	85%	67%	69%	3%	0%
Niranjan et al. [58]	2010	46	13	62 ^	71%	78%	NR	78%	0%	0%
Hasegawa et al. [59]	2010	97	11.4 ^	68	64%	69%	60%	NR	NR	5%
Yu et al. [60]	2000	38	8-18 *	16 ^	90%	NR	NR	NR	NR	0%
Chung et al. [61]	2000	31	12.2 ^	36 ^	87%	NR	NR	NR	0%	3%

* Range; ^ Mean; abbreviations: FU = follow-up; Gy = gray; No = number; NR = not reported; PFS = progression-free survival; y = year.

## Data Availability

No new data were created or analyzed in this study. Data sharing is not applicable to this article.

## Supplementary Materials

The following are available online at https://www.mdpi.com/article/10.3390/cancers13194998/s1; Figure S1: Funnel plots for tumor control/remission; Figure S2: Funnel plots for 5-year progression free survival; Figure S3: Funnel plots for new onset hypopituitarism; Figure S4: Forrest plots for new onset hypopituitarism.
