# Supplementary material for: Gamma Knife Radiosurgery for Pituitary Tumors: A Systematic Review and Meta-Analysis
Source: Cancers (Basel). 2021 Oct 5;13(19):4998. doi: 10.3390/cancers13194998 (PMC8508565; doi:10.3390/cancers13194998)
Supplement: Supplementary file 1 [file cancers-13-04998-s001.zip › cancers-1393681-supplementary.pdf]

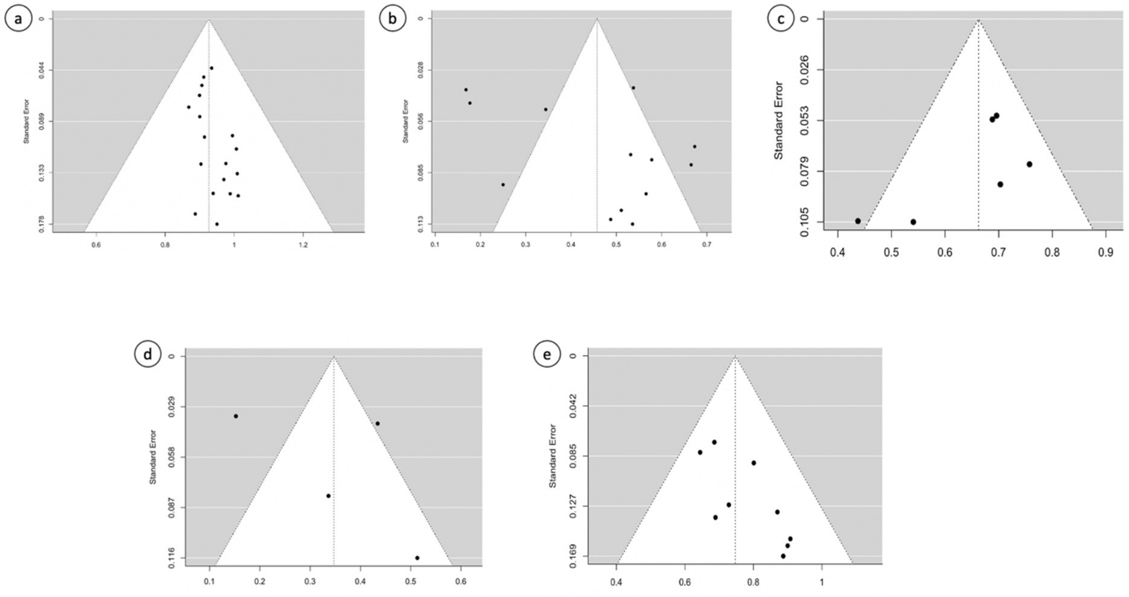

Figure S1. Funnel plots for tumor control/tumor remission with observed proportion outcome on x-axis and standard error on the y-axis. Tumor control for non-functioning pituitary adenomas **(a)**; tumor remission for growth-hormone pituitary adenomas **(b),** adrenocorticotropic hormone-secreting pituitary adenomas **(c)** and prolactin hormone-secreting pituitary adenomas **(d)**; tumor control for craniopharyngiomas **(e).**

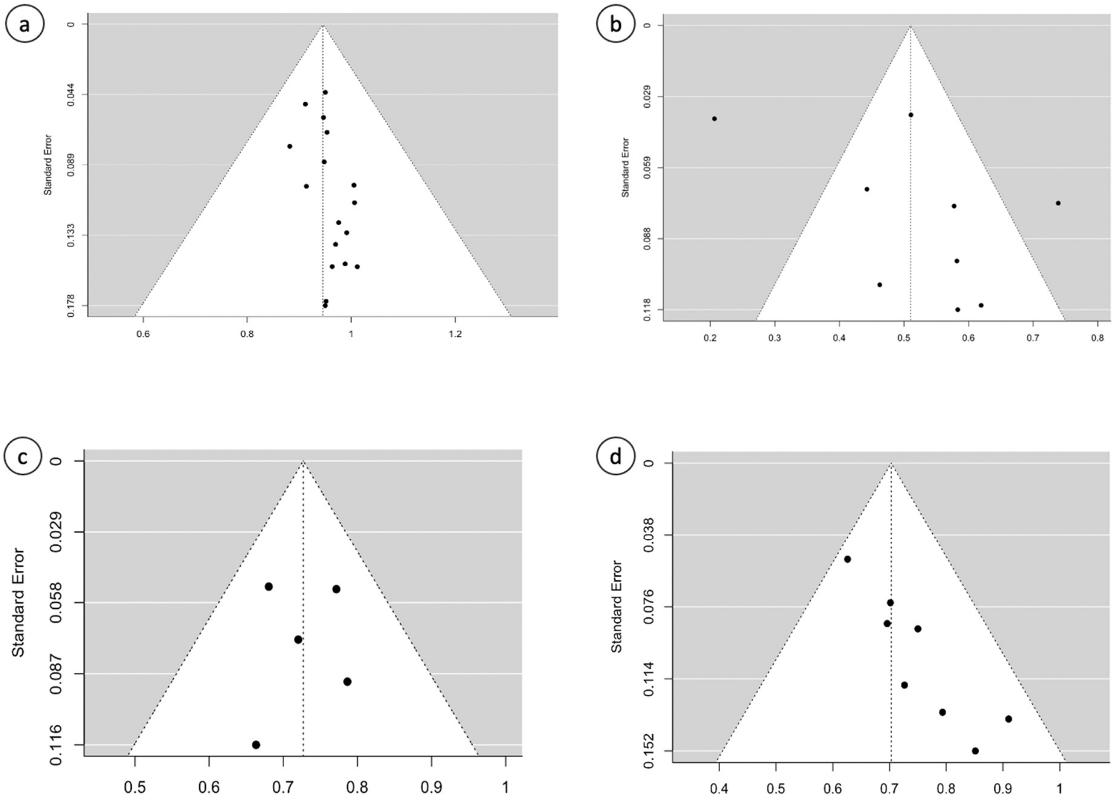

Figure S2. Funnel plots for 5-year progression free survival with observed proportion on x-axis and standard error on the y-axis. 5-year progression free survival for non-functioning pituitary adenomas **(a)**, for growth-hormone pituitary adenomas **(b),** adrenocorticotropic hormone-secreting pituitary adenomas **(c)** and for craniopharyngiomas **(d).**

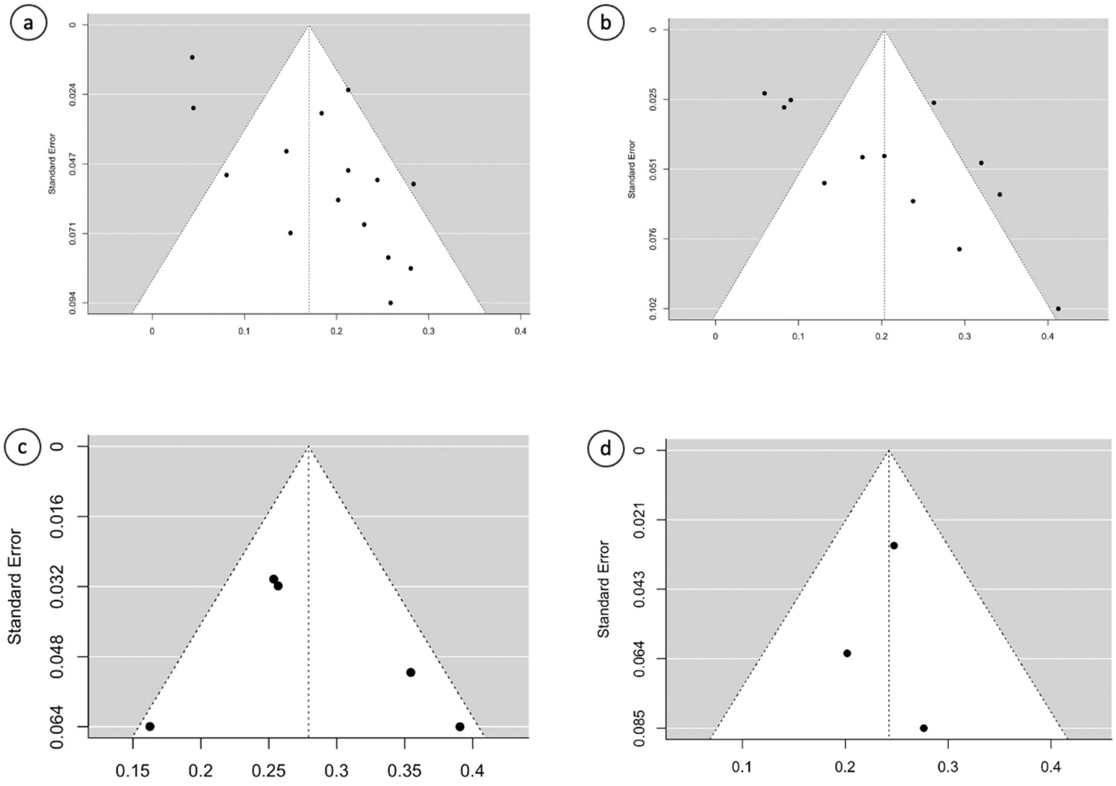

Figure S3. Funnel plots for hypopituitarism with observed proportion on x-axis and standard error on the y-axis. New onset hypopituitarism for non-functioning pituitary adenomas **(a)**, for growth-hormone pituitary adenomas **(b),** adrenocorticotropic hormone-secreting pituitary adenomas **(c)** and for prolactinomas **(d).**

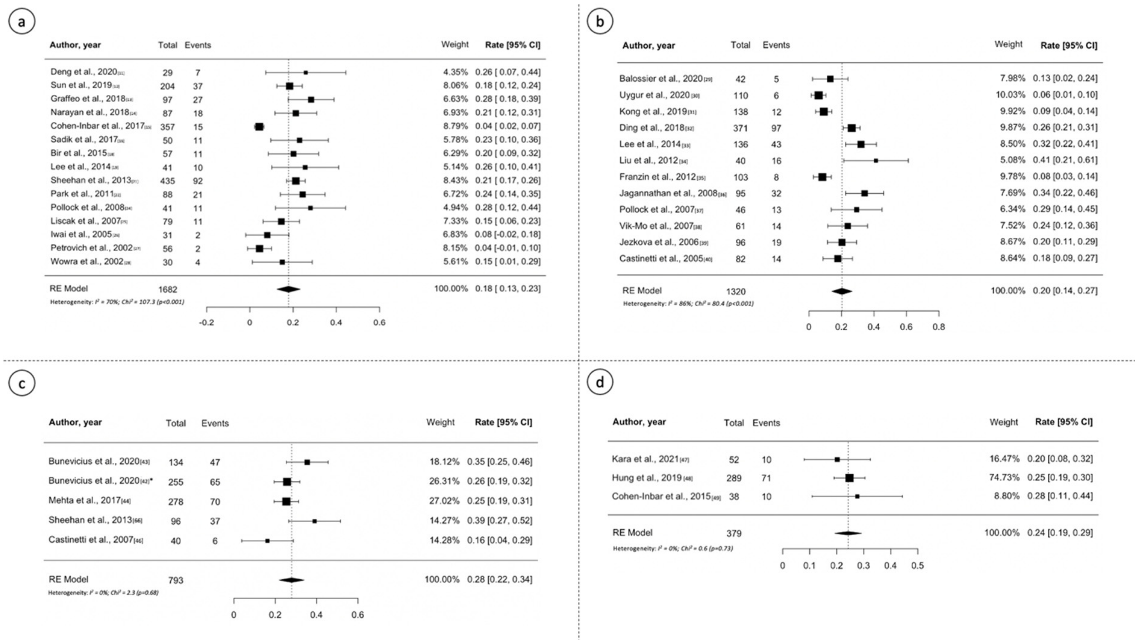

Figure S4. Forest plots of new onset hypopituitarism following Gamma Knife treatment for non-functioning pituitary adenomas **(a)**, growth-hormone pituitary adenomas **(b),** and adrenocorticotropic hormone-secreting pituitary adenomas **(c)** and prolactin hormone-secreting pituitary adenomas **(d)**.
